# Use of End-of-Class Quizzes to Promote Pharmacy Student Self-Reflection, Motivate Students to Improve Study Habits, and to Improve Performance on Summative Examinations

**DOI:** 10.3390/pharmacy8030167

**Published:** 2020-09-10

**Authors:** Ruth Vinall, Eugene Kreys

**Affiliations:** 1 Department of Pharmaceutical & Biomedical Sciences, College of Pharmacy, California Northstate University, Elk Grove, CA 95826, USA; 2 Department of Clinical & Administrative Sciences, College of Pharmacy, California Northstate University, Elk Grove, CA 95826, USA; ekreys@cnsu.edu

**Keywords:** formative assessment, self-reflection, exam performance

## Abstract

Underperforming students are often unaware of deficiencies requiring improvement until after poor performance on summative exams. The goal of the current study was to determine whether inclusion of individual end-of-class formative quizzes, which comprise of higher level Bloom’s questions, could encourage students to reflect on and address deficiencies and improve academic performance. Ninety-seven out of 123 first-year pharmacy students (79%) enrolled in a Biochemistry and Cell & Molecular Biology course participated in a single-blinded, randomized, controlled, crossover study. Paired *t*-test analyses demonstrated that that implementation of individual end-of-class formative quizzes resulted in significantly higher summative exam scores for below average students (*p* = 0.029). Notably, inclusion of quizzes significantly improved performance on higher Bloom’s questions for these students (*p* = 0.006). Analysis of surveys completed by students prior to summative exam indicate that the formative end-of-class quizzes helped students identify deficiencies (89%) and making them feel compelled to study more (83%) and attend review sessions (61%). Many students indicated that quizzes increased stress levels (45%). Our collective data indicate that quizzes can improve summative exam performance for below average first year pharmacy students, and improve self-reflection and student motivation to study. However, the impact on student stress levels should be considered.

## 1. Introduction

Formative assessments support learning by helping students better understand course expectations, promoting engagement and discussion, and promoting self and peer reflection [1,2,3]. They also allow students and instructors to gauge competency prior to summative examinations and can thereby allow for corrective actions to be put in place if gaps in knowledge and understanding are identified. Formative assessments may be completed by individual students or groups of students, and can include presentations, discussion boards, reflection papers, and quizzes. The assessment type and format used is typically based on the course learning objectives and/or student level.

Formative assessments are a key part of team-based learning (TBL) pedagogy, an active learning-based pedagogy which is increasingly being used to teach medical profession degree courses, including in pharmacy schools [4,5,6,7,8,9]. In TBL, formative assessments first occur at the beginning of each module; individual and team readiness assurance tests (iRATs and tRATs) are employed to assess students’ knowledge and understanding of pre-class assignments. Typically, iRAT/tRATs comprise of lower Bloom’s level questions. Significant class time is then dedicated to formative assessments designed to further build competency, with student teams, typically comprised of 4–7 students, being asked to work on application exercises consisting of higher Bloom’s level questions. While focus is placed on ensuring that all students engage in the team application exercises, a major concern is some students may not be able to achieve individual competency through completion of higher level Bloom’s formative assessments completed solely as a team. Likewise, this lack of competency may not be recognized by the student and/or the instructor prior to summative assessments. This concern extends to any teaching format which depends on team-based formative learning activities and assessments to help students achieve course learning objectives.

The primary goal of the current study was to determine whether including end-of-class individual formative assessments would improve individual student performance on summative assessments by allowing students, in particular lower-performing students, to better recognize and address gaps in their ability to achieve course learning objectives following completion of formative team assessments. We also assessed the impact of including individual end-of-class formative assessments on student course satisfaction, including stress, and confidence levels.

## 2. Materials and Methods

### 2.1. Study Design

**Figure 1:**
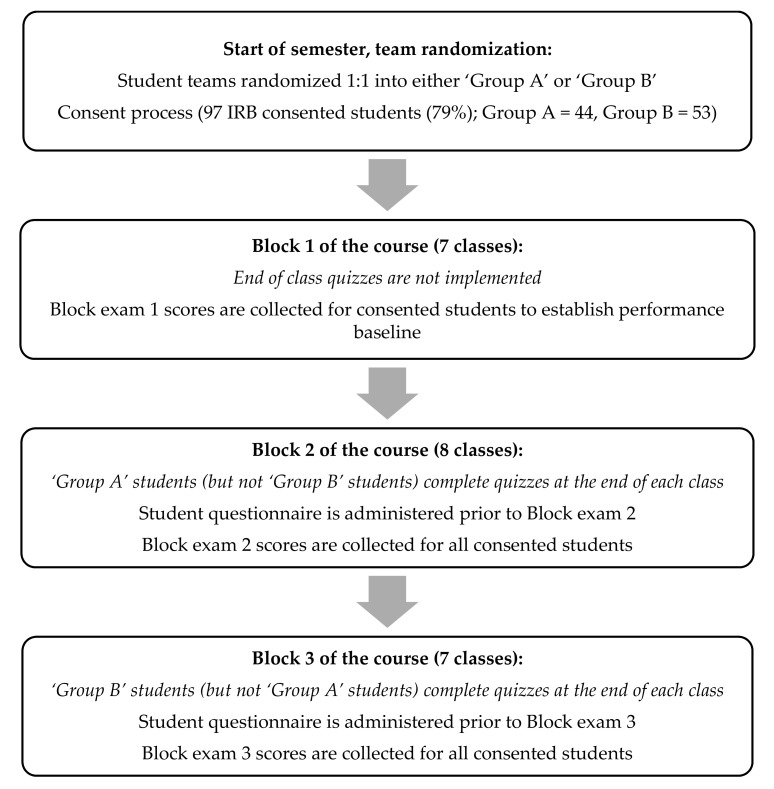
Study plan. This was a single-blinded, randomized, controlled, cross-over study. A total of 97 students consented to participate and were randomly assigned to ‘group A’ or ‘group B’. No end of class quizzes were administered during block A. Quizzes were implemented during blocks 2 and 3 of the course for ‘group A’ and ‘group B’, respectively. This cross over design strategy allowed for comparison of the impact of taking quizzes versus not taking quizzes on student exam performance while effectively increasing n for the study.

This was a single-blinded (course instructors were blinded to student participation), randomized, controlled, crossover study. The study was approved by the California Northstate University (CNU) IRB (approval #0516-01-001) prior to implementation. A flow chart which provides a summary of the study design is shown in Figure 1. All students of a first-year pharmacy course enrolled in the biochemistry and Cell & Molecular Biology course at California Northstate University College of Pharmacy (CNUCOP) were invited to participate in the research study. A USD 10 gift card was provided to students who consented to participate and completed study surveys. TBL pedagogy was used for this course, with each team comprised of 5–6 students. Students were preassigned to specific teams by the college’s administration using the usual process independent of study participation. Teams were randomized to ‘group A’ or ‘group B’ in a 1:1 ratio and all team members, regardless of whether or not they agreed to participate in the research study, were required to complete end of class quizzes assigned to their team. Research data was only collected and assessed for consented students. Surveys were only administered to students who consented to participate in the study. The course comprised of three blocks, each of which culminated with a summative assessment block exam) covering the material taught within the corresponding block. Formative team assessments (team application exercises) were employed throughout the course.

During the first block, no individual end-of-class formative assessments (end-of-class quizzes) were administered to either groups of students and a typical TBL pedagogy format was followed; individual readiness assurance test (iRAT) at the start of class followed by a mini-lecture then team activities and class discussion. The first block exam was used to identify high performing students (defined as those scoring in the top 50th percentile) and low performing students (defined as those scoring in the lower 50th percentile). During the second block, at the conclusion of each class, students within ‘group A’ were required to complete an end-of-class quiz comprising of higher-level Bloom’s questions (Bloom’s level 2 or above per revised Bloom’s taxonomy [10]) on the topics covered during the team application exercises that day. Student within ‘group B’ did not complete block 2 end-of-class quizzes. The quiz questions along with the answers were however made available to the entire class the following day regardless of study participation or group allocation to help ensure that no student was placed at a disadvantage in terms of the totality of the material made available for block exam preparation. This was felt to be an important aspect of the study, not only to determine the independent effect of end-of-class quizzes (which were closed book, timed, proctored, and graded), beyond the delivery of extra preparation material, which can be provided through various modalities, but also for ethical reasons whereby the participation in the study or team allocation resulted in disparate level of the available educational resources. For the third block, the crossover was executed and the roles between ‘group A’ and ‘group B’ were reversed, whereby students in ‘group B’ completed end-of-class quizzes and students in ‘group A’ did not. It is noteworthy that all end-of-class quizzes were graded and contributed to a small portion (2%) of the overall course grade. A survey primarily consisting of ten five-point Likert scale questions was administered to all students prior to block 2 and 3 exams in order to measure the student experience in terms of identification of knowledge gaps, exam preparation, and class satisfaction (Table 1). The survey also requested students to predict their letter grade (A (90.0–100%), B (80.0–89.9%), C (70.0–79.9%), D (60.0–69.9%), E (below 60%)) reflecting their eventual performance on the block exam.

### 2.2. Statistical Analysis

For the primary evaluation, paired analyses were used to compare student performance on exams corresponding to the blocks where students received end-of-class quizzes and exams corresponding to the blocks where students did not receive end-of-class quizzes. Each student’s block 2 and 3 exam results were transformed into z-scores so as to provide a standardized measure of student performance on block exams. Due to the nature of z-scores it was essential to stratify the results to allow for comparisons to be made. Analysis was stratified based on the low and high-performing students as designated by the results of block one examination. Therefore, separately among low and high performing students, a paired *t*-test or Wilcoxon-signed rank test was chosen to compare z-scores between student performance on exams covering the material of blocks corresponding to receiving and not receiving end-of-class quizzes. Subgroup analysis based on the complexity of block exam question as defined by Bloom’s taxonomy of lower level recall questions (Bloom’s level 1) and higher-level understanding and application questions (Bloom’s level 2 and above) was conducted. Pearson correlation or Spearman Rho were used to analyze the association between end-of-class quiz performance and student performance on exam scores. A Shapiro-Wilk test was used to determine normality of exam results and determine the selection of parametric or non-parametric tests for analysis. The survey results were presented in a descriptive manner. McNemar test was used to compare each individual student’s ability to predict the letter grade on the block exam covering the material of blocks corresponding to receiving and not receiving end-of-class quizzes.

## 3. Results

A total of 97 out of 123 students consented to participate in this study (79%). Forty-four of these students belonged to the teams that were randomized to ‘group A’, while 53 belonged to teams that were randomized to ‘group B’. Block 1 exam data was used to establish baseline academic performance in the course prior to administration of individual end-of-class formative quizzes during blocks 2 and 3 of the course. A Shapiro-Wilk test with a *p*-value of 0.176 denoted a normal distribution for the block exam 1 data thereby supporting parametric data analysis. The overall average exam 1 score was 83.9% ± 13.4%, with those in upper 50th percentile scoring an average of 93.4% ± 4.2%, and lower 50th percentile scoring an average of 74.3% ± 12.5%. The block 1 exam proved to be a good tool to differentiate between high performing (upper 50th percentile of students) and lower performing students (lower 50th percentile of students) with a reasonable standard deviation of 13.4%.

A normal distribution was also determined for block exams 2 and 3. Overall, average performance on block 2 and block 3 exams was 81.0% ± 10.9% and 82.7% ± 11.6%, respectively. Average scores for the individual end-of-class formative quizzes were 82.9% ± 10.3% and 83.0% ± 12.1% for Block 2 and Block 3, respectively (note that these are the average scores for consented students, not all students). Correlation analysis revealed an association between average individual end-of-class formative quiz scores and block exam score (R^2^ = 0.17, *p*-value = 0.002); however, only 17% of variation in block exam score can be explained by average end-of-class quiz scores indicating that while the quiz questions may be valid, overall student performance on quizzes is only weakly predictive of block exam performance.

Comparison of exam 2 and 3 z-scores allowed for the impact of individual end-of-class formative quizzes on high versus lower performing students to be assessed. Z-score comparisons for high versus lower performing students on block 2 and 3 summative exams revealed that individual end-of-class formative quizzes had the greatest impact on exam performance in lower performing students, with a particularly improved exam performance on higher level understanding and application questions. For low performing students, administration of end-of-class quizzes resulted in a significant increase of 0.29 ± 0.90 z-scores (*p* = 0.029) on their block exam performance. For high performing students, end-of-class quizzes did not results in a statistically significant change in exam performance with an increase of 0.07 ± 0.78 z-scores (*p* = 0.518).

Z-score comparisons were also used to determine if differences in performance on lower versus higher level Bloom’s exam questions existed for lower versus higher performing students. For lower performing students, subgroup analysis of exam performance limited to lower level Bloom’s questions did not result in a statistically significant change in exam performance with an increase of 0.07 ± 1.18 z-scores (*p* = 0.068), while for higher level understanding and application questions an increase of 0.40 ± 0.98 z-scores (*p*-value = 0.006) was observed. For higher performing students, subgroup analysis based on question type did not reveal statistically significant differences in performance; a decrease of 0.04 ± 1.00 z-scores (*p* = 0.762) was observed for lower level Bloom’s questions, and an increase of 0.10 ± 0.94 z-scores (*p*-value = 0.477) was observed for higher level Bloom’s questions.

Based on the standard deviation of exam scores of around 12%, an overall improvement of 0.29 z-scores in lower performing students and 0.40 z-scores specifically for higher level questions represents an estimated 3.5% and 5.0% increase in exam scores, respectively.

**Figure 2:**
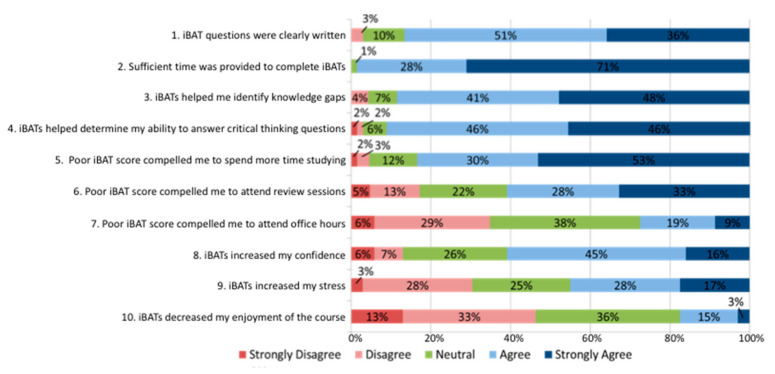
Survey Results. The text of the questions in this figure are paraphrased, full text for each question can be found in Table 1.

Survey data (Figure 2 and Supplementary Table S1) showed that 89% of students strongly agreed or agreed that quizzes helped them identify gaps in knowledge and understanding of key concepts (survey question 3, Table 1). A total of 92% of students strongly agreed or agreed that quizzes helped them determine whether they were able to effectively answer critical thinking questions (survey question 4, Table 1). These data indicate that including quizzes helped to promote student self-reflection. The inclusion of quizzes improved students’ motivation to study; 83% of students strongly agreed or agreed that poor performance on quizzes would make them feel compelled to study more (survey question 5, Table 1), 61% of students strongly agreed or agreed that poor performance on quizzes would make them feel compelled to attend review session (survey question 6, Table 1), and 28% of students strongly agreed or agreed that poor performance on quizzes would make them feel compelled to attend office hours (survey question 7, Table 1). Quizzes also increased student confidence levels; 61% of students strongly agreed or agreed that quizzes increased their confidence levels (survey question 8, Table 1). Survey data demonstrated that there were also negative consequences associated with implementation of quizzes; 45% of students strongly agreed or agreed that quizzes increased stress levels (survey question 9, Table 1), and 18% of students strongly agreed or agreed that inclusion of quizzes decreased course enjoyment (survey question 10, Table 1). An evaluation of a relationship between answers to survey questions to exam performance did not identify a significant association for any of the survey questions. The end-of-class quizzes did not significantly improve the student ability to predict their ability their performance on block exams; students receiving end-of-class quizzes were able to predict their exam performance 46% of the time, as compared to 41% of the time for those students not receiving end-of-class quizzes (*p* = 0.678).

## 4. Discussion

To our knowledge, this is the first study using a crossover trail design to evaluate the effectiveness of formative assessments on student performance. Our data demonstrate that individual formative end-of-class quizzes comprising of higher Bloom’s level questions can improve summative exam performance for lower-performing PharmD students enrolled in a first year pharmacy course. Importantly, quizzes improved performance of these students on higher level Bloom’s summative exam questions, i.e., the type of question lower-performing students often struggle with most. Previous studies have demonstrated formative assessments are beneficial and can improve pharmacy student performance in a variety of didactic and experiential education settings [11,12,13,14], For example, Gums et al. demonstrated that individual formative assessments improved overall student performance in a pharmacy skills laboratory course using historical controls as a comparator [13]. Bullock et al. performed a before versus after study in which they demonstrated use of the muddiest point technique following the first course summative pharmacotherapy course exam improved subsequent formative summative exam performance [14]. These data align with the broader educational literature which has demonstrated formative tests enhance retention and improve educational outcomes, a phenomenon often referred to as the ‘testing effect’ [15,16]. The use of a crossover trail design in our study significantly minimized the contribution of confounding factors, which is especially critical when evaluating an effect of any intervention on educational outcomes considering the various unknown confounding factors that affect variation in student performance over the duration of the course. Likewise, our study is unique in that it specifically assessed the impact on lower-performing pharmacy students. Our finding that individual formative end-of-class quizzes benefit lower-performing students is important because these students are at higher risk of academic failure, an outcome which can have serious consequences for both students and institutions, including increased student stress levels and financial burden, and increased drop-out rates. Identifying and supporting underperforming pharmacy students is clearly of utmost importance, and our data support the inclusion of individual formative end-of-class quizzes as an effective way to do this in this setting.

Our survey data indicate that the including individual formative end-of-class quizzes comprising of higher level Bloom’s questions promoted student self-reflection and motivated students to implement self-directed corrective actions, and it is likely that the increase in performance of lower-performing students that we observed was at least in part due to these individual formative quiz-induced behaviors. In support of this, others have shown that implementation of other strategies which promote self-reflection, including the use scaffolding, can help improve summative exam performance [17,18,19,20,21]. The ability of the quizzes to promote self-reflection and self-directed learning behaviors in first year students is important to emphasize here because these behaviors support life-long learning, a capability that is essential for healthcare professionals. These behaviors are also strongly encouraged and monitored by the US Accreditation Council for Pharmacy Education (ACPE) as part of their review of curricular and co-curricular activities within US pharmacy programs [22,23,24]. Our survey data also indicate that many students found the individual end-of-class formative quizzes stressful. While this is somewhat expected, it is a major concern and should be monitored as others have shown that increasing the number of tests in a course can negatively impact student performance due to increased stress levels [25]. Encouragingly, many students stated that the quizzes increased their confidence levels prior to summative exams and the quizzes did not appear to have a major negative effect on overall course enjoyment.

This study was conducted within a course that uses team-based learning (TBL) pedagogy, and as such the course relied heavily on formative team assessments to support higher level learning. There are multiple advantages to asking pharmacy students to work in teams solve complex problems in a formative assessment setting, e.g., students build their communication and collaboration skills, teamwork promotes student engagement, and discussing problems with others promotes knowledge retention [4,5,6,7,8,9]. If teams are formed properly, then the collective ability level of all teams should be similar, and the TBL format and structure encourages high performing students within the team to help lower performing students. While this very likely occurs in most team-based formative assessments, it is not unusual to encounter some students who state they did not realize they needed additional help answering higher level Bloom’s questions until after taking summative exams. Likewise, some students who perform poorly on summative exams are not identified as being ‘at risk’ by instructors because they performed well on low level Bloom’s iRAT questions prior to summative exams. Our finding that end-of-class individual quizzes comprising of higher level Bloom’s questions improved performance of lower-performing students suggests that team-based formative assessments may not adequately prepare all students for summative exams, and indicate that inclusion of quizzes which test individual student higher Bloom’s capabilities prior to summative exams may be warranted when using teaching pedagogies which rely heavily on formative team activities to support higher level learning (e.g., TBL and problem-based learning (PBL)).

## 5. Study Limitations

While a large proportion of the class consented to participate in this study, the study size was still relatively small (total of 97 students). Likewise, this study only included data from a single first year pharmacy course (Biochemistry and Cell & Molecular Biology) at a single college of pharmacy (CNUCOP) and as such it is not certain that similar results would be observed if individual end-of-class formative quizzes were implemented within other courses or at other colleges of pharmacy. Also, only one summative assessment was used to stratify students into the below versus above average performance groups and as such it is possible that factors other than academic ability (e.g., students experiencing personal and/or health issues on the day of the exam) could have influenced group assignment and thereby have reduced our ability to see the true impact of end-of-class quizzes on below average student performance. Finally, providing all students with access to the quiz questions and answers prior to summative block exams, regardless of their assigned group, likely limited our ability to observe the general impact of formative assessments that were taken during class time. Rather, by isolating the effect of individual participation in end-of-class formative quizzes, which were administered during class time, on summative exam performance, we were able to measure the effectiveness of this specific intervention to improve student learning.

## 6. Conclusions

In conclusion, our data support the use of individual end-of-class formative quizzes to help improve summative exam performance for lower-performing pharmacy students in the setting described and similar settings, and indicate these quizzes encourage student self-reflection and self-directed learning behaviors. The impact of quizzes on student stress levels is a major consideration and should be monitored. Lastly, the implementation of individual end-of-class formative quizzes can significantly increase faculty workload and this is also an important consideration.

## Figures and Tables

**Table 1:** Survey questions.

	Survey Questions
1	The quiz questions were clearly written and understandable
2	Sufficient time was provided to complete the quizzes
3	The quizzes helped me identify gaps in knowledge and understanding of key concepts
4	The quizzes helped me determine whether or not I can effectively answer critical thinking-type questions relating to each topic
5	If I received a score of less than 70% on a quiz I would feel compelled to spend more time studying the related topic
6	If I received a score of less than 70% on an quiz I would feel compelled to attend review sessions
7	If I received a score of less than 70% on an quiz I would feel compelled to attend office hours
8	The quizzes have increased my confidence levels
9	The quizzes have increased my stress levels
10	The quizzes have decreased my enjoyment of this course

## Supplementary Materials

The following are available online at https://www.mdpi.com/2226-4787/8/3/167/s1, Table S1: Survey data.
