# Supplementary material for: Use of End-of-Class Quizzes to Promote Pharmacy Student Self-Reflection, Motivate Students to Improve Study Habits, and to Improve Performance on Summative Examinations
Source: Pharmacy (Basel). 2020 Sep 10;8(3):167. doi: 10.3390/pharmacy8030167 (PMC7558579; doi:10.3390/pharmacy8030167)
Supplement: Supplementary file 1 [file pharmacy-08-00167-s001.pdf]

Supplementary Table S1: Survey data.

Question	Strongly Disagree	Disagree	Neutral	Agree	Strongly Agree
1. The quiz questions were clearly written and understandable	0	3	10	51	36
2. Sufficient time was provided to complete the quizzes	0	0	1	28	71
3. The quizzes helped me identify gaps in knowledge and understanding of key concepts	0	4	7	41	48
4. The quizzes helped me determine whether or not I can effectively answer critical thinking-type questions relating to each topic	2	2	6	46	46
5. If I received a score of less than 70% on a quiz I would feel compelled to spend more time studying the related topic	2	3	12	30	53
6. If I received a score of less than 70% on an quiz I would feel compelled to attend review sessions	5	13	22	28	33
7. If I received a score of less than 70% on an quiz I would feel compelled to attend office hours	6	29	38	19	9
8. The quizzes have increased my confidence levels	6	7	26	45	16
9. The quizzes have increased my stress levels	3	28	25	28	17
10. The quizzes have decreased my enjoyment of this course	13	33	36	15	3
